# Recent advances in understanding and managing hypoparathyroidism

**DOI:** 10.12688/f1000research.22717.1

**Published:** 2020-07-23

**Authors:** Mishaela R. Rubin

**Affiliations:** 1 Department of Medicine, Metabolic Bone Diseases Unit, Division of Endocrinology, Vagelos College of Physicians & Surgeons, Columbia University, 180 Fort Washington Ave, New York, NY, 10032, USA

**Keywords:** hypoparathyroidism

## Abstract

Hypoparathyroidism is a rare endocrine disorder which leads to hypocalcemia, hypercalciuria, and hyperphosphatemia. Complications include nephrocalcinosis with renal dysfunction, reduced quality of life, and abnormal skeletal properties. Conventional therapy with calcium and vitamin D analogs addresses hypocalcemia but has important limitations. Parathyroid hormone (PTH) therapy is a fundamental advance, although the effects of PTH on long-term complications require additional testing. Continuous PTH therapy is likely to be particularly advantageous for addressing renal, quality of life, and skeletal complications. Overall, much progress has been made, yet more information is needed to improve our understanding and management of hypoparathyroidism.

## Introduction

Hypoparathyroidism is a rare endocrine disorder in which the production of parathyroid hormone (PTH) by the parathyroid gland is absent or inappropriately low ^1, 2^. The prevalence of hypoparathyroidism in the United States and Europe is estimated at 23–37 per 100,000 individuals ^3, 4^. The most common cause of hypoparathyroidism is damage to or removal of the parathyroid glands during neck surgery, accounting for approximately 75% of cases ^2, 4^. Autoimmunity targeting the parathyroid gland and, rarely, genetic disorders can also lead to hypoparathyroidism ^1, 5^. DiGeorge syndrome, the most common genetic cause, is associated with a microdeletion in chromosome 22q11.2 and parathyroid hypoplasia ^6, 7^. Other genetic causes include autosomal dominant hypocalcemia (ADH), in which an activating mutation of the calcium-sensing receptor (CaSR) decreases its set-point ^8^, and autoimmune polyendocrine syndrome type 1, in which a mutation in the autoimmune regulator gene ( *AIRE*) leads to destruction of the parathyroids and other endocrine glands ^9^.

PTH plays a fundamental role in mineral homeostasis by promoting renal reabsorption of calcium and stimulating renal phosphate excretion. It also promotes conversion of 25-hydroxyvitamin D to 1,25-dihydroxyvitamin D (1,25[OH] _2_D), the active form of vitamin D, which increases the absorption of calcium and phosphate from the gastrointestinal tract. Moreover, PTH is a powerful regulator of bone turnover, such that PTH deficiency leads to decreased bone turnover. Thus, patients with hypoparathyroidism experience hypocalcemia, hyperphosphatemia, hypercalciuria, reduced levels of 1,25[OH] _2_D, and abnormally low bone turnover, resulting in overly mineralized bone ^1, 2, 10, 11^.

The diagnosis of hypoparathyroidism requires confirmed hypocalcemia in the presence of undetectable or inappropriately low levels of endogenous PTH and the absence of hypomagnesemia, the latter condition being a reversible cause of hypoparathyroidism; other potentially reversible causes include iron or copper overload and autoimmune causes ^1^. Hypocalcemia typically manifests as perioral numbness, paresthesias, neurocognitive deficits, weakness, and carpopedal muscle spasms, although potentially life-threatening complications, such as cardiac arrhythmias, laryngeal spasm, tetany, and seizures, can also occur ^1, 2, 12, 13^.

## Conventional therapy

Conventional management of hypoparathyroidism consists of oral calcium (e.g. calcium carbonate or citrate) and active vitamin D treatments (e.g. calcitriol), as well as thiazide diuretics to increase calcium reabsorption at the distal tubule, and magnesium supplementation as needed. The goal is to maintain serum calcium levels just below or within the lower normal range. Additional goals include preserving a normal level of serum phosphate, preventing an elevation of the serum calcium-phosphorus product, minimizing hypercalciuria, and avoiding kidney stones and mineralization of soft tissues.

Although conventional therapy increases intestinal calcium absorption and corrects hypocalcemia, it does not replace other functions of PTH and, in the absence of PTH’s calcium-retaining effect in the kidney, can lead to hypercalciuria ^1, 2, 10, 14– 16^. Other limitations of conventional therapy include unpredictable occurrences of hypocalcemia and hypercalcemia, increased calcium-phosphorus product, and long-term complications such as ectopic calcifications, nephrocalcinosis, nephrolithiasis, and renal dysfunction ^1, 2, 10, 14– 16^. Symptoms of hypocalcemia and reduced quality of life (QoL) are common among patients managed with conventional treatment ^17– 20^.

## PTH therapy

Treatment of hypoparathyroidism with intermittent PTH injections has been an important advance. Studies by Winer *et al*. with PTH(1-34) showed that subcutaneous injection could maintain normocalcemia in hypoparathyroid adults ^21^ and children ^22^ as well as calcitriol for as long as 3 years ^23, 24^ and that twice-daily dosing reduced the total amount of PTH required ^25^. Recombinant human PTH [rhPTH(1-84)] is full-length PTH that was approved in the United States in 2015 as a once-daily subcutaneous administration as an adjunctive treatment of adults with hypoparathyroidism who could not be well controlled on conventional therapy alone ^26^. Safety and efficacy were demonstrated in placebo-controlled and open-label studies ^27– 30^. In the pivotal REPLACE study, a 24-week, double-blind, placebo-controlled, randomized phase III study conducted with 134 patients, 53% of patients receiving rhPTH(1-84) versus 2% of patients receiving placebo met the primary study end point (≥50% reduction in calcium and calcitriol doses with maintenance of normal serum calcium) ^28^.

## Renal complications

Without the renal calcium-retaining effects of PTH, conventional therapy often leads to elevated urinary calcium excretion and long-term renal complications, including nephrocalcinosis, nephrolithiasis, and, consequently, the development of chronic kidney disease ^15, 16^. In a case-control study, using the Danish National Patient Registry, Underbjerg and colleagues found decreased eGFR (<60 mL/minute) in 21% of hypoparathyroid (n = 431, mostly postsurgical) patients ^31^. This increased risk of renal disease was significantly associated with a higher number of hypercalcemic episodes and a higher calcium-phosphate product ^31^. An even higher rate of kidney disease was shown in a retrospective chart review of 120 mostly postsurgical hypoparathyroid patients at a tertiary care center ^15^. This report showed two- to 17-fold greater rates of developing chronic kidney disease (stage 3–5) than age-appropriate adjusted rates, with 41% of hypoparathyroid patients having chronic kidney disease ^15^. Specific risk factors for a lower eGFR included age, duration of disease, and proportion of time with relative hypercalcemia ^15^. Nonsurgical hypoparathyroid patients were also shown to experience renal disease, with 14% out of 165 patients having an eGFR <60 mL/minute; a younger age at presentation and a higher serum calcium-phosphorus product increased the risk for nephrocalcinosis ^32^. Taken together, these studies show that renal disease is a frequent consequence of hypoparathyroidism and is likely mediated by exposure to hypercalcemia and abnormal calcium-phosphate homeostasis. It is unknown whether reducing serum phosphate levels, either by diet or phosphate binders, minimizes renal complications.

PTH treatment, by increasing renal calcium reabsorption, would be expected to decrease urinary calcium excretion in hypoparathyroidism. Disappointingly, this effect has not been demonstrated in a RCT. In the REPLACE study, in both rhPTH(1-84) and placebo groups, urinary calcium excretion declined from baseline, but, at week 24 in both groups, the mean urinary calcium excretion remained above the upper limit of normal for women and near the upper limit of normal for men ^28^. However, the rhPTH(1-84) group had significant reductions in serum phosphate and the serum calcium-phosphate product throughout the study compared with patients receiving placebo ^33^.

In the 60-month open-label extension of REPLACE (n = 40), the mean urinary calcium level declined into the normal range for men and women ^30^. The serum phosphate and calcium-phosphate product also declined, while serum calcium, creatinine, and eGFR remained unchanged ^30^. Similar beneficial effects on urinary calcium excretion were seen in the other long-term open-label study of rhPTH(1-84), in which urinary calcium excretion declined significantly over 8 years ^27^. In that study, serum phosphate and the calcium-phosphate product did not decrease but remained within the normal range, as did eGFR. Overall, these long-term uncontrolled studies suggest that urinary calcium excretion and serum phosphate levels remain decreased with prolonged rhPTH(1-84) therapy. Intuitively, reduced urinary calcium excretion would be expected to decrease the risk of renal dysfunction in hypoparathyroid patients; rigorously controlled clinical trials are required to validate this hypothesis.

## Quality of life complications

Hypoparathyroid patients typically suffer from symptoms related to increased neuromuscular excitability, such as parasthesias and carpopedal spasm. In addition, hypoparathyroid patients often describe neurocognitive complaints, including anxiety, depression, fatigue, and various cognitive deficits ^13, 18, 34, 35^. They have lower QoL scores, using validated tools, mostly the Short Form Health Survey 36 (SF-36), a self-administered 36-item questionnaire, compared with reference norms ^18, 19, 34, 36, 37^. Both mental and physical domains are affected, although the mechanisms for the deficits are uncertain. It may be that the physical complaints are attributable to reduced muscle function ^34^, which might in turn be associated with fluctuations in serum calcium. The mental QoL complaints might be explained by effects of calcium on the central nervous system, as supported by findings in primary hyperparathyroidism of neuropsychological symptoms ^38^. Interestingly, QoL seems to be more reduced in patients with postsurgical hypoparathyroidism compared with non-surgical hypoparathyroidism ^39^. This might be explained by postsurgical patients also having hypothyroidism ^39^. Alternatively, patients with life-long nonsurgical hypoparathyroidism might be less sensitive to changes in their QoL, having never experienced a full sense of well-being.

The effects of PTH treatment on QoL are unclear. Anecdotally, patients report marked improvements, but a significant favorable effect of PTH-replacement therapy was not shown in double-blind randomized trials compared with controls receiving conventional therapy ^20, 36^. In the REPLACE study, SF-36 scores did not differ between the two treatment arms, rhPTH(1-84) versus placebo, at 24 weeks ^36^. One can speculate that this negative finding was due to the lack of sustained PTH elevations with the frequency of PTH administration used. Specifically, once-a-day rhPTH(1-84) injections can cause fluctuations over the 24-hour period ^40^, with an initial rise leading to transient hypercalcemia and possible symptoms of nausea, poor concentration, and polyuria, followed by a fall in serum calcium levels with possible hypocalcemia symptoms.

Open-label uncontrolled data regarding PTH treatment and QoL are more positive ^41^. In a long-term open-label study of 28 hypoparathyroid patients treated with rhPTH(1-84) therapy over 8 years, improvement using the SF-36 was observed, particularly in subjects with impaired SF-36 scores at baseline and in those whose requirements for conventional therapy decreased substantially ^19^. It is possible that PTH itself, rather than restoration of eucalcemia, may improve QoL. Support for this idea includes the observation that PTH can cross the blood–brain barrier ^42^ and can stimulate PTH2 receptors that are in the brain ^43^. These PTH2 central nervous system receptors have been associated with areas involved in the regulation of depression and anxiety in animals ^44, 45^.

Importantly, a disease-specific questionnaire is not available for hypoparathyroidism. Most studies of PTH effects on QoL used the SF-36, which is subjective and does not adequately measure cognitive deficits, fatigue, physical endurance, or muscle strength. A single instrument that can characterize and measure the burden of disease experienced by patients with hypoparathyroidism is needed. A subjective “Hypoparathyroidism Symptom Diary” is being developed and tested ^46^. This is a 13-item instrument, which assesses symptoms including muscle cramping, tingling and muscle spasms/twitching, fatigue, and cognition, as well as anxiety, sadness, and depression, and impacts on sleep, ability to exercise, ability to work, and family relationships ^46^. The development of a validated tool to assess disease burden and efficacy of treatment in hypoparathyroidism will be a key advance.

## Skeletal complications

Hypoparathyroidism is associated with low bone turnover (the process of coupled bone formation and bone resorption), which is associated with increased bone mineral density and abnormal bone microarchitecture ^47, 48^. The reduction in bone formation is demonstrated by the decrease of tetracycline labelling in bone biopsies compared with controls ^49^. Notably, fracture risk in hypoparathyroidism is uncertain. Case-control studies demonstrate no differences in overall hypoparathyroidism fracture rates ^16, 35^, while other reports show an increased fracture risk at the spine and upper extremities ^13, 15, 50^.

With rhPTH(1-84) therapy, open-label data show an initial large increase in bone turnover markers at 1 year of therapy, followed by a new steady state that remains above baseline with continued therapy ^30, 51^. In an 8-year study of rhPTH(1-84), lumbar spine bone mineral density increased in the early years and plateaued after 4 years of rhPTH(1-84) while an increase in total hip bone mineral density was seen in the later years of PTH therapy. Femoral neck bone mineral density remained unchanged throughout the study, while one-third radial bone mineral density declined modestly ^27^. The increase at the trabecular-enriched lumbar spine and the decrease at the cortical 1/3 radius were reminiscent of the patterns observed with intermittent PTH treatment for osteoporosis. Histomorphometric analysis of bone biopsies showed that intra-trabecular tunneling was present, with increases in cancellous bone volume and trabecular number and cortical porosity ^52^, suggesting exuberant stimulation of bone remodeling. Fracture data are needed to determine whether bone fragility is present in the context of long-term rhPTH(1-84) use.

## Continuous PTH administration

As noted above, daily administration of rhPTH(1‐84) did not demonstrate a reduction in hypercalciuria compared with conventional therapy in the REPLACE registration RCT ^28^. This shortcoming might have been due to the relatively short half-life of rhPTH(1‐84) with daily subcutaneous administration. PTH levels are transiently high after injection but then drift downwards during the hours preceding the subsequent injection ^53^. Without continuous PTH exposure to stimulate renal calcium reabsorption, urinary calcium excretion increases.

The benefits of continuous PTH exposure were shown in pump studies by Winer and colleagues. Continuous subcutaneous infusion of PTH(1‐34) with an insulin pump to hypoparathyroid patients mimicked endogenous PTH secretion more closely than intermittent PTH injections ^54, 55^. In both hypoparathyroid adults and children, the pump delivery method led to normalization of serum calcium, urinary calcium excretion, and bone remodeling as measured by bone turnover markers. Moreover, serum phosphate levels normalized in adult hypoparathyroid patients after continuous subcutaneous infusion of PTH(1‐34), although not in hypoparathyroid children. Magnesium levels improved significantly in adults and children. Notably, continuous infusion of PTH(1‐34) was superior by all measures compared with a twice-daily subcutaneous dosing regimen with PTH(1-34), despite a >60% lower daily dose of PTH with infusion ^54, 55^. These studies suggest that continuous exposure to PTH provides a highly physiologic effect to reverse the biochemical derangements that are a hallmark of hypoparathyroidism.

An important gap in the management of hypoparathyroid patients is the lack of a technology to continuously monitor endogenous calcium levels. Given that calcium fluctuations can be triggered by exercise, general illness, or unpredictable causes ^1^, hypoparathyroid patients would benefit from knowing their calcemic variability in real time, giving them a chance to adjust their treatment individually and dynamically. A device for monitoring calcium to allow for dose adjustments in response to fluctuations in blood calcium would facilitate such titration. Such a continuous calcium sensor in combination with PTH delivery by a pump device could constitute a transformative closed-loop “artificial parathyroid” system for hypoparathyroid patients.

## Current and future therapies

rhPTH(1-84), or Natpara, was recalled in the United States in September 2019 owing to a potential problem: over the course of treatment, the rubber septum of the Natpara cartridge is punctured by a needle to obtain the daily dosage, and when this occurs repeatedly, there is a possibility that small rubber particles may detach from the septum into the cartridge ^56^. A “Special Use Program” is available to patients previously prescribed Natpara who are at extreme risk of life-threatening complications as a result of discontinuation of treatment. This program requires that product usage be limited to a single dose per cartridge, instead of 14 doses per cartridge, to minimize the potential of particle formation caused by repeat punctures ^56^. Importantly, PTH cessation can lead to rebound hypocalcemia, likely due to the reversal of the enlarged remodeling space favoring bone formation as the skeleton returns to a low turnover state ^57^.

Off-label use of teriparatide (rhPTH[1-34]) has been used in hypoparathyroid patients. Although it is not FDA-approved for the treatment of hypoparathyroidism, there is experience with its use in clinical trials ^21– 25^. Individualized dosing, administered as twice-daily or even thrice-daily injections, might be necessary because of the relatively short effect of teriparatide to raise serum calcium levels ^58^.

A potential new therapy is TransCon PTH, an inactive prodrug of PTH(1-34) that is designed to achieve an infusion-like profile by liberating active PTH in a sustained fashion ^59^. PTH(1-34) is transiently bound via a linker to a chemically inert carrier that shields PTH from binding to the PTH type 1 receptor (PTHR1) and prolongs the peptide’s circulation half-life due to reduced renal clearance ^59^. In euparathyroid and hypoparathyroid rats and in euparathyroid monkeys, it led to steady concentrations of PTH within the normal range, with persistent increases in serum calcium and decreases in serum phosphate levels ^59^. The results of a phase I trial with TransCon PTH in healthy volunteers demonstrated a markedly prolonged half-life of free PTH of ~60 hours ^60^. Notably, a net increase in bone resorption with TransCon PTH administration has been observed ^59^. Although this could theoretically lead to a primary hyperparathyroidism-like profile, increased resorption could have a salutary effect to decrease the high bone mineral density and increase the low bone turnover rate of hypoparathyroidism. A phase II trial with TransCon PTH is on-going.

An additional potential therapy is PCO371, a novel orally active compound that acts as an agonist of PTHR1 ^61^. In a series of *in vitro* and *in vivo* experiments, Tamura *et al*. demonstrated that oral PCO371 has similar biochemical and renal effects as PTH injections, particularly in hypoparathyroid rats, restoring serum calcium levels and lowering serum phosphate levels without increasing urinary calcium excretion ^61^. Although it was less potent than human PTH (hPTH)(1-34) *in vitro*, the *in vivo* effects were more long-lasting than those of hPTH(1-34) and hPTH(1-84), consistent with greater bioavailability. PCO371 is currently undergoing clinical testing. If effective, this oral PTH analogue could overcome concerns about injectable use and be a future treatment option for hypoparathyroid patients.

A further creative approach may be a long-acting analogue of PTH (LA-PTH). This modified peptide mediates prolonged and enhanced effects to increase blood calcium levels in hypoparathyroid rats as compared to PTH(1-34) and PTH(1-84) ^62, 63^. The prolonged signaling action appears to involve a mechanism of pseudo-irreversible binding to a distinct PTHR1 conformation (R ^0^), which maintains high affinity for the ligand through multiple rounds of G protein coupling ^64^. The biological actions of LA-PTH in humans remain to be investigated.

## Conclusion

Our knowledge of hypoparathyroidism has increased over the past few decades, yet unresolved questions remain. It is unknown whether the long-term complications of hypoparathyroidism can be prevented or reversed. Moreover, key tools are lacking, such as a validated disease-specific tool to measure QoL and a continuous calcium sensor to enable real-time dose titration. PTH therapy is likely to be most effective when administered in a physiologic manner that minimizes serum and urinary calcium fluctuations, yet large and long-term studies testing continuous PTH exposure are lacking. Answers to these and other key questions will ultimately improve our understanding and management of this rare disease.
